# The use of stool specimens reveals *Helicobacter pylori* strain diversity in a cohort of adolescents and their family members in a developed country

**DOI:** 10.1016/j.ijmm.2017.11.005

**Published:** 2018-03

**Authors:** Brendan Dolan, Lucy Burkitt-Gray, Stephen Shovelin, Billy Bourke, Brendan Drumm, Marion Rowland, Marguerite Clyne

**Affiliations:** a School of Medicine, University College Dublin, Dublin, Ireland; b Conway Institute of Biomolecular and Biomedical Science, University College Dublin, Dublin, Ireland; c The National Childrens Research Centre, Crumlin, Dublin, Ireland

**Keywords:** *Helicobacter pylori*, Fecal specimens, Genotyping, Biprobe assay, qPCR

## Abstract

*Helicobacter pylori* infection occurs within families but the transmission route is unknown. The use of stool specimens to genotype strains facilitates inclusion of complete families in transmission studies. Therefore, we aimed to use DNA from stools to analyze strain diversity in *H. pylori* infected families. We genotyped *H. pylori* strains using specific biprobe qPCR analysis of *glmM, recA* and *hspA*. Concentration of *H. pylori* organisms before DNA isolation enhanced subsequent DNA amplification. We isolated *H. pylori* DNA from 50 individuals in 13 families. T_m_ data for at least 2 of the 3 genes and sequencing of the *glmM* amplicon were analyzed. Similar strains were commonly found in both mothers and children and in siblings. However, 20/50 (40%) individuals had multiple strains and several individuals harbored strains not found in other family members, suggesting that even in developed countries sources of infection outside of the immediate family may exist. Whether infection occurs multiple times or one transmission event with several strains occurs is not known but future studies should aim to analyze strains from children much closer to infection onset. The presence of multiple stains in infected persons has implications for antibiotic sensitivity testing and treatment strategies.

## Highlights

• Concentration of *H. pylori* in feces before DNA isolation enhances DNA amplification.
• Analysis of DNA in feces allows for H. pylori transmission studies in communities.
• Multiple strains are commonly found in infected individuals.
• Family members can harbor strains not found in other family members.
• Infection with multiple strains has implications for antibiotic sensitivity testing.

## 1 Introduction

*Helicobacter pylori* colonizes the gastric mucosa of humans and induces a complex inflammatory response with the development of chronic antral gastritis in both children and adults (Blanchard and Czinn, 2017, McColl, 2010, Suerbaum and Michetti, 2002). A small proportion of those infected will develop peptic ulcer disease and gastric cancer (Leow et al., 2016, Sonnenberg, 2013). While *H. pylori* was considered one of the commonest bacterial infections worldwide, there has been a rapid decline in the prevalence of *H. pylori* over the last 100 years in developed countries, with an accompanying decline in the incidence of *H. pylori* associated peptic ulcer disease and gastric cancer (Lanas and Chan, 2017, Leow et al., 2016, McColl, 2010, Suerbaum and Michetti, 2002).

*H. pylori* infection almost always occurs in childhood, and persists for life unless specifically treated with antimicrobials (Leow et al., 2016, McColl, 2010, Rowland et al., 2006, Suerbaum and Michetti, 2002). There is no known reservoir of *H. pylori* outside the human stomach. While infection is clustered in families (Drumm et al., 1990), there is little information on the intra-familial factors which facilitate transmission. Some studies suggest that transmission between siblings is more likely than parent to child transmission (Cervantes et al., 2010), however, parents are an obvious source of infection for initial colonization of children in a family. Despite the serious consequences of *H. pylori* infection, particularly in countries with a high prevalence of gastric cancer, we still do not know how transmission occurs and what factors might disrupt transmission.

Potential transmission routes include oral-oral, gastric-oral or fecal-oral. Isolation of *H. pylori* from stool has rarely been reported (Kelly et al., 1994, Parsonnet et al., 1999). Reports of culture from the oral cavity are rare and a number of studies have suggested that the presence of *H. pylori* in the oral cavity is transient (Ferguson et al., 1993, Hirsch et al., 2012, Mapstone et al., 1993, Olivier et al., 2006). Furthermore epidemiological data do not support oral–oral transmission as co-habiting couples are infected with different strains; and treated adult patients are not infected by their untreated infected partner (Cutler and Schubert, 1993). A number of studies support the potential role of gastroenteritis in the transmission of *H. pylori* (Janzon et al., 2009, Perry et al., 2006). Parsonnet et al. demonstrated that *H. pylori* can be cultured easily from induced emesis in volunteers (Parsonnet et al., 1999) and on occasion from stool specimens folowing the use of cathartics (Parsonnet et al., 1999).

Studies of transmission that rely on upper gastrointestinal endoscopy to obtain gastric biopsies are limited by the number of asymptomatic family members or complete households that can be included (Krebes et al., 2014). The use of stool specimens for isolation of DNA from *H. pylori* to genotype strains present in different family members and conduct transmission studies is an attractive option as it facilitates the inclusion of large numbers and complete families. DNA detection circumvents the potential strain selection bias of culture based techniques. However, a particular challenge associated with using stool specimens is the low concentration of *H. pylori* DNA compared to that of other gastrointestinal organisms.

Puz et al. (Puz et al., 2008) described a novel genotyping protocol for *H. pylori* in stool specimens using biprobe qPCR assays and fragments of *glmM* and *recA* genes as target sequences. When strains had identical melting points they sequenced the *glmM* amplicon to confirm strain identity. The discriminatory capacity of the method was 100%. They reported clonal identities in 9/10 European families and 7/8 African households.

Between 1997 and 2001 we prospectively evaluated the incidence of *H. pylori* in a cohort of Irish families (Rowland et al., 2006). Young age (<5 yrs) was the single biggest risk factor for acquisition of infection and older children did not get infected despite living in households with infected parents and siblings (Rowland et al., 2006). From this cohort we identified a number of families who currently are infected with *H. pylori* (using the carbon-13 labeled urea breath test (^13^C UBT) and collected stool specimens from each of them with the aim of isolating *H. pylori* DNA from their feces and using this DNA to investigate the relatedness of strains within the families.

## 2 Materials and methods

### 2.1 Participants and sample collection

The families who participated in this study have been described previously (Rowland et al., 2006). Briefly, between 1997 and 2002 317 index children and their families were enrolled in the first prospective study of the acquisition of *H. pylori* infection in childhood (Rowland et al., 2006). The index children were between 24 and 48 months of age at enrolment and had ^13^C UBTs carried out at baseline and annually thereafter for 4 years. 290 index children and their families completed the full follow-up. 28 index children (8.56%) were infected at baseline in 2007 and over the remaining 4 years of follow-up a further 20 children became infected with *H. pylori*. The incidence of *H. pylori* infection was highest among the 2–3 year olds (5.05 per 100 person years of follow-up, 95% CI 1.6-11.8) and declined progressively as children got older (Rowland et al., 2006).

In 2013 we traced 250/290 (86.2%) index children and their families and invited them to participate in a second follow-up study. The *H. pylori* status of families was again determined using the ^13^C UBT as described elsewhere (Rowland et al., 1997). Families in which the index child and at least 1 other member were infected were invited to provide a fecal/stool sample. Participants were provided with stool collection kits and instructions on how to collect a sample of stool. Fresh stool samples were collected and transported to the laboratory within 3 h and either processed immediately or frozen at −20 °C until use.

### 2.2 ^13^C urea breath test

The ^13^C UBT was performed as described previously (Rowland et al., 1997). After a 2 h fast breath samples were collected at baseline and 30 min after ingestion of 75 mg ^13^C urea. A cut-off of 5.0 ^13^CO_2_ 0/00 was used to classify participants as infected with *H. pylori*. To ensure that the *H. pylori* status of all participants was accurately determined in 2013, participants with (i) a borderline ^13^C UBT value (2.5–6.0 over baseline); (ii) a result different from 2002; or (iii) a new positive result (younger sibling not previously tested) had their breath tests repeated after an overnight fast. The result of the test performed after an overnight fast was defined as the correct result.

### 2.3 Ethics

Approval for the study was provided by the Irish College of General Practitioners. All participants over 18 years of age provided signed informed consent for the study while those under 18 yrs of age provided assent and their parents/guardians provided signed consent for their participation in the study.

### 2.4 Bacterial strains and culture

*H. pylori* strains J99, 26695, P12, N6, PU4, PU44, G27 and SS1 were routinely cultured at 37 °C on Columbia Blood Agar base (Oxoid) supplemented with 7% (vol/vol) defibrinated horse blood under microaerophillic conditions generated using CampyGen gas packs (Oxoid). *Listeria monocytogenes* EGDe was cultured at 37 °C on Brucella broth (Oxoid) supplemented with 1% (wt/vol) Agar (Oxoid). *Salmonella typhimurium* SL1344 and *Escherichia coli* HB101 were cultured at 37 °C on Luria-Bertani Agar (Oxoid).

### 2.5 DNA isolation from cultured bacteria

Following culture of bacteria biomass from one plate was collected into sterile PBS (Oxoid). The OD_600_ was adjusted to ∼1 and 1 ml of bacteria was collected by centrifugation. The pellet was resuspended in 567 μl TE buffer (Sigma-Aldrich). Following the addition of 30 μl 10% (vol/vol) SDS and 3 μl proteinase K (20 mg/ml; Qiagen), the bacterial suspension was incubated for 1 h at 3 °C. Following incubation, 600 μl of phenol/chloroform/isoamyl alcohol (25:24:1; Invitrogen) was added, the solution was mixed well and centrifuged at 14,000 x *g* for 5 min at 4 °C. The aqueous layer was transferred to a fresh tube, an equal volume of chloroform (Sigma-Aldrich) was added, mixed well and centrifuged as before. The aqueous layer was transferred to a fresh tube and 2 volumes of ethanol (Sigma–Aldrich) and 1/10 volume 3 M sodium acetate (Sigma–Aldrich) were added, mixed well and incubated on ice for 30 min. Eluted DNA was pelleted by centrifugation for 15 min at 4 °C. The pellet was washed in 70% ethanol, air dried for 15 min and resuspended in 50 μl TE buffer at 4 °C.

### 2.6 Isolation of DNA from fecal material

A number of methods were used to isolate DNA from fecal material including the CTAB (cetyl trimethylammonium bromide) method of DNA isolation and the QIAamp DNA Stool Mini Kit (Qiagen). In addition, we developed an antibody capture method to enrich for *H. pylori* organisms present in stool followed by use of the PowerFecal DNA isolation kit (Mo Bio Laboratories). For the CTAB method 30 μl of 10% (wt/vol) SDS and 3 μl proteinase K (20 mg/ml) was added to 200 mg of fecal material resuspended in 567 μl of Tris EDTA (TE) buffer (10 mM Tris; 1 mM EDTA, pH 8.0) and the suspension was incubated for 1 h at 37 °C. Following incubation 100 μl 5 M NaCl and 80 μl CTAB/NaCl (100 mM Tris-HCl (pH 7.5), 25 mM EDTA, 1.5 M NaCl, 2% (wt/vol) CTAB, and 0.3% (vol/vol) β-mercaptoethanol) was added and, the sample was incubated for 10 min at 65 °C. An equal volume of phenol/chloroform/isoamyl alcohol (25:24:1) was added, and after centrifugation the aqueous layer was transferred to a fresh tube and an equal volume of chloroform/isoamyl alcohol (24:1; Sigma-Aldrich) was added. After centrifugation the aqueous layer was transferred to a fresh tube and DNA was eluted with isopropanol. DNA was washed with 70% ethanol, air dried and resuspended in 100 μl TE buffer. DNA was also isolated from 200 mg of fecal material using the QIAamp DNA Stool Mini Kit (Qiagen) as described in the manufacturer’s instructions (pathogen detection protocol).

### 2.7 DNA isolation from fecal material following antibody enrichment for *H. pylori*

A 48 well plate (Nunc) was coated overnight with a polyclonal antibody against *H. pylori* (Dako). The antibody was diluted to 15 μg/ml in bicarbonate buffer pH 9.2. 150 μl of antibody solution was added to each well and the plate was incubated overnight at 4 °C. The plate was blocked with blocking buffer (0.1 M phosphate buffer pH 7.2, 150 mM NaCl, 1% (wt/vol) BSA and 0.5% (vol/vol) Tween 20) for 90 min at room temperature. 250 mg of fecal material was homogenized in 1 ml of antigen buffer (0.1 M phosphate buffer pH 7.2, 150 mM NaCl) using a TissueLyser II (30 Hz/s for 40 s; Qiagen). Following homogenization the sample was transferred to a well on the antibody coated plate and then incubated overnight at 4 °C on a shaking platform. Unbound material was removed and DNA was isolated from the bound material using the PowerFecal DNA isolation kit (Mo Bio Laboratories) according to the manufacturers instructions. 750 μl of Bead Solution was added to the well containing bound material and was mixed well by pipetting. 60 μl of solution C1 was added, mixed well and incubated for 10 min at 60 °C. Following incubation the suspension was transferred to a Dry Bead Tube. The tubes were vortexed at maximum speed for 10 min using a bead beater. Tubes were centrifuged at 13,000 × g for 1 min and the supernatant transferred to a clean 2 ml Collection Tube. 250 μl of Solution C2 was added and the tube was vortexed briefly to mix. Following incubation at 4 °C for 5 min tubes were centrifuged again. Up to 600 μl of supernatant was transferred to a clean 2 ml Collection Tube and 200 μl of Solution C3 was added and tube was vortexed briefly. After incubation at 4 °C for 5 min tubes were centrifuged again for 1 min and the supernatant transferred to a clean 2 ml Collection Tube. 1200 μl of Solution C4 was added and the contents of the tube mixed by vortexing for 5 s. 650 μl of supernatant was loaded onto a Spin Filter which was centrifuged at 13,000 × g for 1 min. The flow through was discarded. 500 μl of Solution C5 was added and following centrifugation for 1 min at 13,000 × g the flow through was discarded. The Spin Filter was placed in a clean 2 ml Collection Tube. 100 μl of Solution C6 was added to the center of the filter membrane. Following centrifugation at 13,000 × *g* for 1 min the DNA was collected and stored at −20 °C.

### 2.8 PCR

Gene fragments of the housekeeping genes *ppa,* and *yphC,* the virulence gene *vacA,* and the *H. pylori 16S* gene were amplified by touchdown PCR. Each reaction contained 5 μl 5 x Q5 reaction buffer (New England Biolabs), 0.5 μl 10 mM dNTPS (Bioline), 1.25 μl 10 μM forward and reverse primers, 1 μl 20 mg/ml BSA (Molecular biology grade, New England Biolabs), 0.25 μl Q5 High-fidelity DNA polymerase (New England Biolabs), 13.75 μl molecular biology grade water and 2 μl template DNA. Primers were designed to amplify approximately 500 bp internal fragments of the above genes previously defined in the *H. pylori* multi locus sequence typing scheme (MLST; http://pubmlst.org/helicobacter/). Primers were designed using Primer3 (Untergasser et al., 2012) and their specificity to *H. pylori* was confirmed using BLAST. PCR primer sequences are detailed in Table 1. The amplification program consisted of 98 °C for 3 min, 20 cycles (98 °C for 30 s, annealing temperatures ranging from 65 °C to 55 °C for 30 s with a decrease of 0.5 °C from one cycle to next, 72 °C for 30 s), 40 cycles (98 °C for 30 s, 60 °C for 30 s, 72 °C for 30 s) followed by a final extension at 72 °C for 7 min. The PCR products were separated using a 2% agarose gel, stained with ethidium bromide and visualized under a UV light.

**Table 1:** Primers used for PCR in this study.

Gene	Forward Primer	Reverse Primer	Product Size bp	Source
*ppa*	AGCCATGACGCTRAKYCTTTRAKYCTTT	CTCTTTGTTTTCAAACCCCTTG	490	Osaki et al. (2013)
*vacA*	CCCGATAAAGTTTGGCGCAT	CGTGGCGCCATCATAAAGAG	498	This study
*yphC*	ATTGCGATTTTAGGCCAGCC	CGCACTCAATACCGCATCAA	468	This study
*16S*	TCGGAAGTGGAGCCAATCTT	GGAACGTATTCACCGCAACA	119	Horemans et al. (2011)

Y = C, R = A or G, K = G or T.

### 2.9 qPCR assays targeting *glmM*, *recA* and *hspA*

The housekeeping genes *glmM* (Kansau et al., 1996, Kivi et al., 2003, Raymond et al., 2008) and *recA* (Kuipers et al., 2000, Maggi Solca et al., 2001) previously have been used in the analysis of *H. pylori* strain diversity. Puz et al. have described a qPCR based biprobe method to explore sequence diversity in these genes (Puz et al., 2008). We looked to increase the discriminatory ability of this assay through the analysis of another housekeeping gene. *H. pylori* heat shock protein A (HspA) is a 13 kDa protein of the GroES class. HspA is composed of two domains, the A domain is highly conserved and is homologus to other bacterial heat shock proteins. The carboxyl B domain is a unique 27 amino acid histidine rich domain involved in binding Ni^2+^ (Suerbaum et al., 1994). Sequence variation in the B domain of *hspA* previously has proven useful in the study of *H. pylori* geographical clustering (Ng et al., 1999) as well as the analysis of *H. pylori* strains present within families (Raymond et al., 2004). A 133 bp fragment of the 3′ end of *hspA* was amplified and the sequence variation was analyzed using a Cy-5 labeled DNA probe. PCR primers and the Cy-5 labeled probe were designed using Primer3 and their specificity for *H. pylori* was confirmed using BLAST. For amplification of *glmM, recA* or *hspA* gene fragments the reaction mixture was prepared as follows; 2 μl LightCycler 480 SYBR Green I Master (Roche Diagnostics), 4 μM MgCl2 (*glmM*; New England Biolabs) or 2 μM MgCl2 (*recA* and *hspA*), 0.075 μM forward primer, 0.75 μM reverse primer 0.4 μM Cy5 probe, 2 μl template DNA and the volume was made up to 20 μl with PCR grade water (Roche). Primer and Cy5 probe sequences are detailed in Table 2. Each reaction began with a preliminary denaturation for 10 min at 95 °C, followed by 70 cycles of denaturation at 95 °C for 10 s, annealing at 60 °C (*glmM*), 63 °C (*recA*) or 55 °C (*hspA*) for 10 s and extension at 72 °C for 6 s with a single fluorescence acquisition step at the end of each cycle. Following amplification thermal analysis of the probe-amplicon duplex consisting of 95 °C for 5 s, cooling to 40 °C for 1 min before the temperature was increased to 97 °C at a rate of 0.06 °C/s with continuous fluorescence acquisition followed by a final cooling step at 40 °C for 10 s. Light emission was monitored simultaneously using the 465–510 and 465–660 filter combination of the LightCycler 480 instrument (Roche Diagnostics). Melting curves were constructed automatically by the LightCycler 480 Software (version 1.5.1.62) and were analyzed in the 465–660 channel of the instrument. All samples were run in triplicate.

**Table 2:** PCR primers and Cy5 probes used for Real-Time analysis.

Gene	Forward primer	Reverse primer	Cy5 Probe	Product size bp	Source
*glmM*	TCTAAAAACGCCCTTTCTTCTCA	ATTCGCTCACAAACTTATCCCC	Cy5-CAATTGTCGCTACAAACATGAGCA-biotin	130	(20)
*recA*	GGATATGGGCGATCAGCA	CGGTTGTGGTCTCTGGACTC	Cy5-GTTAAGGAAAATCACGGTGTCTTGCATAAGA-biotin	164	(20)
*hspA*	ACAGCAAGATTCATGCTCTT	CAGAAATCGTTTTAGACGGCA	Cy5-TGGTCATGATTACCTGTATGACAAC-biotin	134	This study

### 2.10 Spiking of fecal material with *H. pylori*

In order to investigate the ability of the biprobe assay to detect and differentiate between *H. pylori* strains we spiked 250 mg of fecal material from uninfected individuals with approximately 5 × 10^4^ *H. pylori* 26695 (Strain 1*), H. pylori* P12 (Strain 2) or a combination of both strains which had been cultivated for 48 h on Columbia blood agar. Spiked samples were stored at −20 °C overnight and *H. pylori* DNA was isolated using the antibody enrichment method described above. Isolated DNA was then examined using the qPCR biprobe assay.

### 2.11 Sequencing

qPCR products were separated using a 2% agarose gel, stained with ethidium bromide and visualized under a UV light. PCR products were excized from the gel using a sterile scalpel blade and DNA was extracted from the gel fragments using Zymoclean™ Gel DNA Recovery Kit (Zymo Research) as per manufacturer’s instructions. Sequencing of purified DNA fragments was performed using the Value Read service from Eurofins Genomics. Obtained sequences were aligned and compared using Clustal Omega (Sievers et al., 2011).

## 3 Results

### 3.1 Protocol development for *H. pylori* specific DNA isolation from stool specimens

**Fig. 1:**
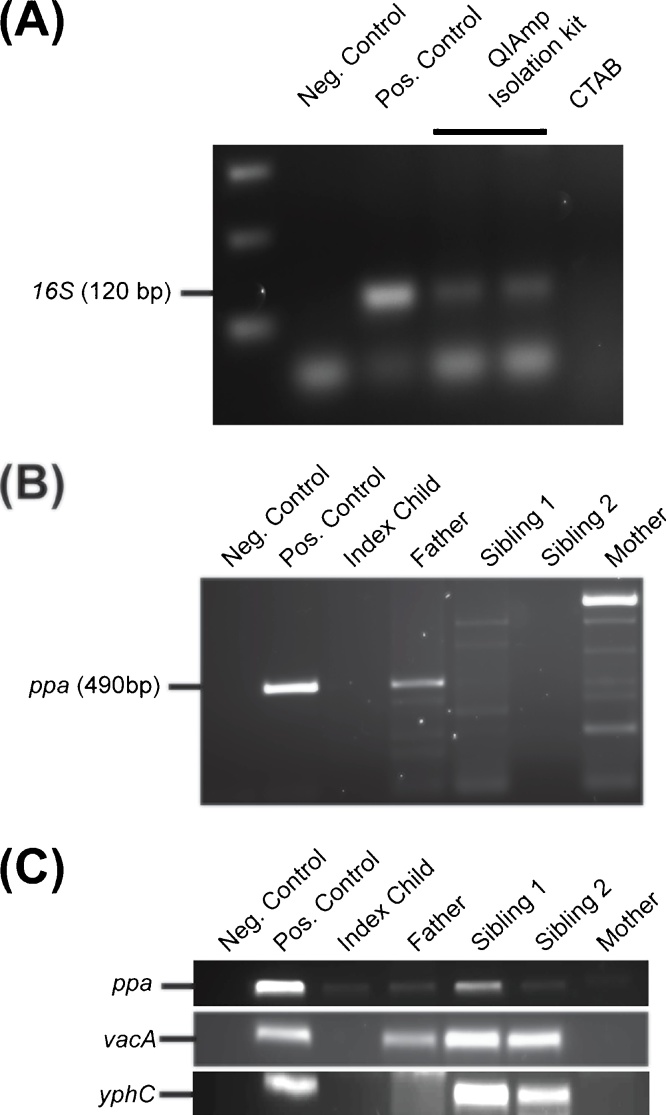
Amplification of *H. pylori* specific products from DNA isolated from stool specimens. (A) PCR amplification of *H. pylori 16S* gene using DNA isolated with the QIAamp DNA stool Mini Kit or CTAB DNA isolation protocol. (B) PCR amplification of *H. pylori ppa* gene following DNA isolation by QIAamp DNA Stool Mini Kit. (C) PCR amplification of *H. pylori* MLST housekeeping genes using DNA isolated by antibody capture based technique and PowerFecal DNA Isolation Kit (Mo Bio). No DNA was added to the PCR reaction for the negative control and genomic DNA isolated from *H. pylori* cultured on Columbia blood agar was used as a positive PCR control.

Initially we used both the QIAamp DNA Stool Mini Kit and the CTAB procedure to isolate DNA from the stool of an *H. pylori* infected adult. Using DNA isolated with the QIAamp DNA Stool Mini kit we could amplify a 120 bp fragment of the *H. pylori* 16S rRNA gene. However we could not amplify *H. pylori* DNA with DNA isolated using the CTAB method (Fig. 1A)

DNA was isolated from fecal material of five *H. pylori* infected members of one family using the QIAamp DNA Stool Mini Kit and PCR was performed to amplify gene fragments ranging in size from 468 to 504 bp, from housekeeping genes used for MLST analysis (Table 1). A 490 bp *ppa* product was clearly amplified from the DNA isolated from an infected father and a faint band was amplified from the DNA isolated from the mother but not with DNA isolated from two children in the family (Fig. 1B). In addition, multiple non-specific PCR products were amplified on some occasions (Fig. 1B). Lack of amplification of a *ppa* fragment with DNA from all infected individuals suggested that the concentration of *H. pylori* DNA in the stool samples was very low and that there was a need to evaluate other methods to isolate *H. pylori* specific DNA from the stool specimens. Therefore we aimed to develop a DNA isolation protocol that recovered bacterial DNA of suitable quality for the reliable amplification of *H. pylori* housekeeping genes.

An antibody capture based technique to enrich for *H. pylori* organisms present in fecal slurry prior to isolation of DNA was used. Previous studies have reported efficient DNA extraction from clinical specimens, including stool, containing multiple microbial species when a bead beating step was included (Halstead et al., 2013, Leite et al., 2013, Yu and Morrison, 2004). Therefore we evaluated the use of the PowerFecal^®^ kit (Mo Bio) which includes a bead beating step for mechanical lysis of the cells in the sample. *H. pylori* DNA isolated using the modified protocol resulted in improved amplification of fragments of *H. pylori* MLST housekeeping genes (Fig. 1C). However, products could still not be amplified from DNA isolated from all infected individuals with all primers. In addition analysis of the forward and reverse strand sequences of purified PCR products revealed low sequence identity between forward and reverse reads and conflicting MLST analysis. Representative results from one individual are shown in Table 3. Therefore, although this method allowed for improved detection of *H. pylori* DNA in stool the quality of DNA extracted did not allow for genotyping of *H. pylori* strains in different individuals using conventional PCR based techniques such as MLST.

**Table 3:** Representative results from stool of one individual of MLST analysis for two *H. pylori* housekeeping genes and for the virulence gene *vacA*.

Gene	Forward read	Reverse read	MLST ST Forward/Reverse	Sequence Identity
*ppA*	+	−	No Match	−
*vacA*	+	+	101/101	49.30%
*yhpC*	+	+	181/181	49.06%

+ = read obtained, − no read obtained.

### 3.2 Biprobe genotyping to discriminate between different *H. pylori* isolates

**Fig. 2:**
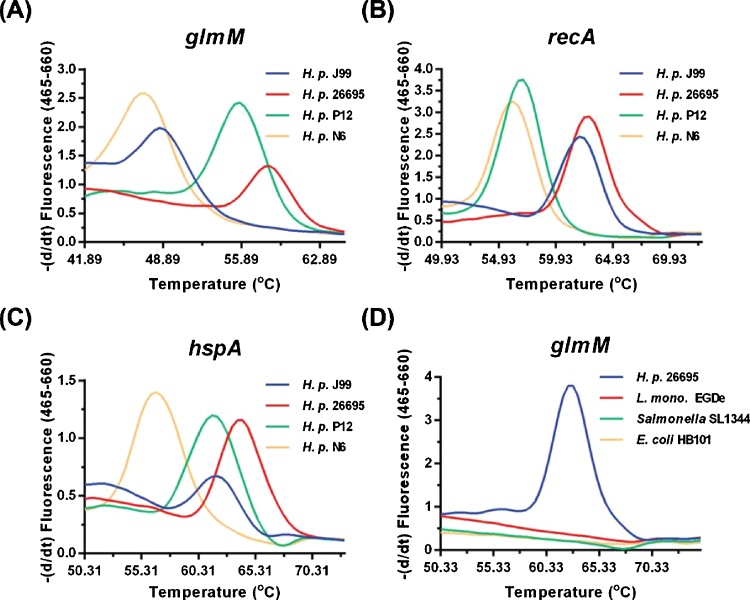
Thermal analysis of probe amplicon duplex in various *H. pylori* strains. Genomic DNA from *H. pylori* strains was examined using the Biprobe assay for three housekeeping genes *glmM* (A and D), *recA* (B) and *hspA* (C). (D) Genomic DNA from *H. pylori* strain 26695 and from other gastrointestinal pathogens was examined using the Biprobe assay for housekeeping gene *glmM.* Detection of *H. pylori* specific DNA only occurred with DNA isolated from *H. pylori*.

As the amplification of *H. pylori* specific sequences was most efficient for short amplicon lengths we next assessed a previously described biprobe qPCR genotyping method to determine if it could discriminate between different *H. pylori* strains present in stool (Puz et al., 2008). This method amplifies short products of 120–180 bp which can subsequently be sequenced. Sequence differences in a short, highly variable region of a PCR amplicon discriminates between strains, with a single-SNP resolution. We used primers and probes targeting *glmM* and *recA,* as previously published (Puz et al., 2008), and also used primers and a probe targeting the B domain of *hspA* in order to improve the ability of the assay to discriminate between different strains present in stool samples. The primer and probes used were able to discriminate well characterized strains of *H. pylori* when DNA from 8 cultured strains was used as template for the PCRs. Representative results with four of these strains are shown in Fig. 2 A-C. Only *H. pylori* DNA was amplified and detected using the primers and probes described and amplification of DNA from other representative gastrointestinal organisms did not occur (Fig. 2D).

**Fig. 3:**
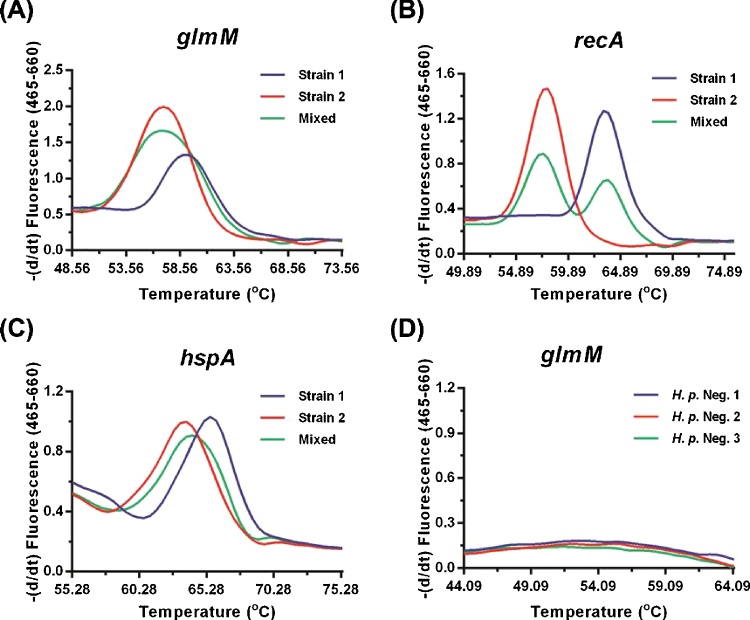
Thermal analysis of probe amplicon duplex in stool specimens spiked with *H. pylori* organisms. Stool specimens from *H. pylori* negative individuals were inoculated with either *H. pylori* strain P12 (strain 1), *H. pylori* strain 26695 (Strain 2), both strains (Mixed) or *H. pylori* strain G27. DNA isolated from inoculated and uninoculated stools was examined using the biprobe assay for three housekeeping genes *glmM* (A and D), *recA* (B) and *hspA* (C). No *H. pylori* specific products were amplified using DNA isolated from stool from 3 *H. pylori* negative individuals.

We then spiked stool samples from an *H. pylori* negative individual with freshly cultured organisms from either a single *H. pylori* strain or from two different strains. Using the biprobe, *H. pylori* DNA isolated from stool samples spiked with organisms from a single strain was detected with each of the three primer pairs and probes (Fig. 3A–C) and there was no signal detected from DNA isolated from stool specimens spiked with organisms other than *H. pylori* (Fig. 3D). When the T_m_s of the *H. pylori* strains were clearly separated it was possible to detect both isolates in a single stool sample using the qPCR biprobe assay (Fig. 3B) but not when there was a significant overlap in the melt curves (Fig. 3A and C). This highlights the importance of investigating more than one gene in order to identify the presence of more than one strain in a sample. However, while we were able to determine T_m_s using the *glmM* and *recA* primers and probes for 100% and 96% (48/50) of the individuals included in the study we could only determine T_m_s for 66% (33/50) of these individuals using the *hspA* primers and probes. We found, in agreement with a previous study (Puz et al., 2008), that sequencing of the *glmM* amplicon greatly enhanced the ability of the assay to differentiate between strains. For example thermal analysis of the *glmM* and *recA* amplicons alone would indicate that the three pairs of spouses included in this study all shared strains. However sequencing of the *glmM* amplicon combined with thermal analysis of both the *recA* and *hspA* amplicons proved that none of the spouses shared a strain (Table 4). Therefore, we proposed the following procedure for the differentiation of *H. pylori* strains present in clinical isolates, first qPCR analysis of at least two but ideally three amplicons (*glmM*, *recA* and *hspA*) combined with sequence analysis of the entire *glmM* amplicon.

**Table 4:** T_m_ for *glmM*, *recA* and *hspA* amplicons and *glmM* sequence alignments in 3 pairs of spouses.

– No product amplified.

*Sequences of amplicons marked with * are provided.

### 3.3 Analysis of clonal lineages in families

Eighty two members of 19 different families, who were known to be infected with *H. pylori* provided stool samples. *H. pylori* DNA was isolated and using the qPCR biprobe assay *glmM*, *recA* and *hspA* were amplified. However for some individuals we could not amplify all three products and/or get reliable sequencing data. T_m_ data for at least two of the three genes and sequencing of the *glmM* amplicon, were generated for 50 individuals from 13 of the 19 families, with a mean family size of 4.61 (range 2–9). For these 13 families we obtained stool samples from the mother, father and children for 3 families, from the father and children for 2 families and from the mother and children for 8 families. The clonal strain identities for these families are outlined in Table 5. As indicated above (Table 4) in the 3 families where stool samples were available from both the mother and the father the strains detected did not share clonal lineage indicating that the parents were not a source of infection for one another. A combination of T_m_ data and *glmM* sequence alignment demonstrated that identical strains were never found in individuals who were from different families.

**Table 5:** Clonal strain identity in families determined using qPCR biprobe and sequencing data.

Family	Mother	Father	C1	C2	C3	C4	C5	C6	C7
12									
Age	60	61	31	28	27	26	24	22	19*
Strain ID	1A	1B	1C,1D	1E	1E	1E	1E	1E	1E
193									
Age	62	61	31	26	19*				
Strain ID	2A, 2B, 2C, 2D	2E, 2F	2A	2A, 2G	2A, 2H				
278									
Age	52	51	20	19*	16				
Strain ID	3A	3B	3A,3C, 3D	3A	3A, 3E, 3F				
70									
Age	53	55	32	27	25	19*			
Strain ID	RX	4A, 4B	4C	4D	RX	4E,4F			
36									
Age	50	50	22	18*					
Strain ID	Neg	5A	5A, 5B	5C					
16									
Age	43		25	22	19*				
Strain ID	6A		6A	6B	6A				
24									
Age	40		20*						
Strain ID	7A		7A, 7B, 7C						
45									
Age	61	57	21	20*	14				
Strain ID	8A	RX	Neg	8A, 8B	8A				
23									
Age	42		23	19*	16				
Strain ID	9A		9B	9C, 9D	9E, 9F				
240									
Age	52	60	19*	18					
Strain ID	10A, 10B	RX	10C, 10D, 10E	10C, 10F					
19									
Age	47		27	19*					
Strain ID	11A		11B	11C					
35									
Age	44		19*	18	5				
Strain ID	12A, 12B		12C, 12D, 12E	Neg	Neg				
83									
Age	42		19*	14	14	9			
Strain ID	13A		13A, 13B	Neg	13A	Neg			

*Index child, Rx individual treated for *H. pylori* infection. C1, C2 etc  =  Child 1, Child 2 etc

In only one family did a child share a strain with the father (Family 36) and in this family the mother had no evidence of *H. pylori* infection (four negative ^13^C UBTs 1997, 2002, 2014, 2014). In 5 of the 13 families there was evidence that each of the children in the family shared a strain with their mother (Families 278, 193, 24, 45, 83). However within each of these 5 families there were individuals who also harbored additional strains that were not detected in other family members. In one family the mother shared a strain with 2 out of 3 infected offspring (Family 16) and in another the mothers strain was detected in none of 7 infected children in the household (Family 12). The oldest child in this house harbored 2 strains that were not detected in any other family member. The six remaining younger children all harbored an identical strain that was not detected in the mother, father or the eldest child (Family 12). In three families no strain common to any family members was detected (Families 23, 19 and 35).

A striking result was that in the majority of families tested multiple strains were detected in at least one member of the family. There were only 2 families where single isolates were detected in all members (Families 16 and 19). Multiple strains were detected in 20 individuals in total (37.7%) (Table 5) and in 2 families multiple strains were detected in all individuals (Families 240 and 35).

## 4 Discussion

The use of the antibody capture technique to capture and concentrate low numbers of *H. pylori* present in stool specimens prior to DNA isolation greatly enhanced subsequent amplification of *H. pylori* specific sequences. Previously immunomagnetic beads coated with a polyclonal *H. pylori* antibody were used to pull out *H. pylori* from stool specimens that had been spiked with the organism (Enroth and Engstrand, 1995). This resulted in the removal of PCR inhibitors and successful amplification of *H. pylori* DNA. In our protocol we did not use immunomagnetic beads but simply coated a multiwell plate with a polyclonal antibody and incubated fecal slurry from *H. pylori* infected individuals in the coated wells. Simple washing removed the fecal material from the well and DNA from the bound bacteria could be isolated and successfully amplified. Thus we demonstrated that an antibody can be used to concentrate *H. pylori* organisms present in stool samples of naturally infected individuals. Others have reported that the number of *H. pylori* organisms required for specific DNA detection in stool specimens is more than 100 times the number required when cultured bacteria are used, and over 10 times the number required when gastric biopsy specimens are used (Giorgio et al., 2016). Improved PCR amplification of specific products following concentration of *H. pylori* organisms in the fecal slurry and inclusion of a bead beating step in the protocol suggests that the number of *H. pylori* organisms present in the stool samples was very low and are likely coccoid in shape and difficult to lyse. The detection rate for *H. pylori* DNA sequences in stool is reported to be approximately 20% lower compared to when gastric biopsy specimens are used (Brennan et al., 2016). The antibody capture technique in addition to concentrating the organisms present in the sample may have also acted to remove potential PCR inhibitors present in the stool specimens. This technique could be adapted to detect the presence of any organism that is present in low numbers in complex biological samples such as feces.

The most efficient and reliable amplification of specific products occurred with primer pairs designed to amplify products less than 200 bp in size. When stools were spiked with freshly cultured organisms the three *H. pylori glmM, hspA* and *recA* specific sequences could be amplified easily. However we could not always amplify all three sequences from DNA isolated from stools of infected individuals. We did not ask individuals to cease taking proton pump inhibitors prior to donation of samples. In addition treatment for infection was based on patient reporting rather than on official medical records or prescription history and this may explain why we were unable to amplify *H. pylori* DNA from some individuals who we knew were infected at the time. It is also likely that the *H. pylori* present in the feces were dead or dying and much of the DNA present was sheared. Difficulties in reliably amplifying high quality PCR products from low abundance bacteria for sequencing suggests that traditional methods such as MLST may not be suitable for genotyping *H. pylori* DNA from stool. Although in this study high resolution melt curve analysis successfully identified sequence differences between model strains cultured in the lab thermal analysis alone was often not sufficient to differentiate between strains present in stools from infected families. Therefore we recommend that sequencing data should be combined with thermal analysis of at least one if not two or more additional genes.

In agreement with previous epidemiological data (Gisbert et al., 2002, Luman et al., 1996, Osaki et al., 2013, Suzuki et al., 1999) our results clearly showed that strains were not shared between spouses and that similar strains were frequently found in mother and child pairs and in siblings. However our results also show that even in families who shared common strains, other strains unique to individual members of the family were identified. In addition 4 families were identified in whom there existed no common strain. The presence of multiple strains in individuals is in keeping with recent whole genome sequencing studies done on isolates cultured from gastric biopsy specimens obtained from individuals living in a developing country. In a whole genome study of 2 families from rural South Africa, Didelot et al. (Didelot et al., 2013) reported, that strains isolated from the antrum and corpus in 4/45 individuals were too distantly related to be derived from each other, providing clear evidence that infection with more than one strain of *H. pylori* can occur. A fifth individual in this study was only 3 years old and while many gene sequences in the two isolates from antrum and corpus were identical there were also a large number of mutation and recombination events detected in other genes which could not have all occurred in the lifetime of this host. Analysis of cultured strains from three generations of a family living in the UK indicated that a highly complex bidirectional exchange of DNA had occurred among the strains (Krebes et al., 2014). There are many possible explanations for the presence of multiple un-shared strains within an individual or families including exchange or recombination of DNA as *H. pylori* adapts to its host, a number of different infective episodes or one transmission episode with multiple strains. Potential sources of different strains from outside of the immediate family include members of the extended family or other infected children (Miyaji et al., 2000, Urita et al., 2013). Unfortunately we did not collect data such as the use of childcare or crèche facilities, involvement of grandparents in child rearing or identification of the primary care giver in the family. Such data should be collected in future studies as it may allow for identification of possible sources of infection from persons outside of the immediate family. It has been suggested that multiple strains occur more frequently in developing countries than in developed countries (Ben Mansour et al., 2016). However our results suggest that the presence of multiple strains in individuals living in developed countries should be further investigated as it has important implications for antibiotic sensitivity testing and for treatment strategies.

In a recent study two individuals with asymptomatic infection were treated with antibiotics and eradication of the infection was confirmed by using ^14^C UBT testing. The individuals were subsequently reinfected with the same strains and genome sequencing was used to analyze the genomes of the two input strains and output strains collected 20 and 44 days after infection. The estimated mutation rate suggested a mutation burst during the acute infection phase that is over 10 times faster than the mutation rate during chronic infection, and orders of magnitude faster than mutation rates in any other bacteria (Linz et al., 2014). Tracking of fluorescent DNA in competent bacteria has shown that *H. pylori* has the capacity to take up large quantities of DNA (Kruger et al., 2016) and natural competence has been shown to promote chronic infection in a murine model of infection (Dorer et al., 2013). The presence of multiple strains combined with natural competence may enable *H. pylori* to adapt to different environmental conditions in the stomachs of individuals.

Whether individuals are infected with multiple strains in a single transmission event or are infected multiple times with single strains cannot be determined from this study. This group of index children were enrolled when they were between the age of 24 and 48 months of age. The majority of those who became infected were infected with *H. pylori* before the age of 3 yrs and the risk of infection after 5 yrs of age was very low despite the presence of other infected family members living in the household (Rowland et al., 1997). In the most recent follow-up of this group of families we demonstrated that among siblings who were not infected in 2002, only one of 165 older siblings became infected (0.6%) after an 11 year follow up. In contrast (9/75) 12% of younger siblings became infected (Rowland et al., in press). Thus we suggest that at least in a developed country such as Ireland, infection only occurs over a short period of time. Stool samples in this study were collected when the majority of participants were over 18 years of age. We do not know if such strain diversity would have been present if *H. pylori* DNA analysis was conducted much closer to the time when children became infected with *H. pylori,* but our data suggests that transmission studies need to be conducted much closer to the onset of infection.

In summary our results show that the use of stool specimens from *H. pylori* infected individuals should facilitate transmission studies in communities. Concentration of *H. pylori* organisms in stool samples prior to DNA isolation greatly enhances subsequent DNA amplification. The presence of multiple strains in infected persons has important implications for interpretation of antibiotic sensitivity test results and for the design of strategies to treat infection. The presence of unique strains in family members suggests that in developed countries sources of infection outside of the immediate family may exist.

## Funding

This work was supported by a grant from the Health Research Board, Ireland (HRA_PHS/2012/23) to MR. Research performed by LBG was supported by The Wellcome Trust (grant no. 105340/Z/14/A). The funders had no role in study design, data collection and interpretation, or the decision to submit the work for publication.

## Conflict of interest statement

The authors would like to declare no conflict of interest.
